# *PGRMC1* Ablation Protects from Energy-Starved Heart Failure by Promoting Fatty Acid/Pyruvate Oxidation

**DOI:** 10.3390/cells12050752

**Published:** 2023-02-27

**Authors:** Sang R. Lee, Moeka Mukae, Kang Joo Jeong, Se Hee Park, Hi Jo Shin, Sang Woon Kim, Young Suk Won, Hyo-Jung Kwun, In-Jeoung Baek, Eui-Ju Hong

**Affiliations:** College of Veterinary Medicine, Chungnam National University, Daejeon 34134, Republic of Korea

**Keywords:** *Pgrmc1*, heart, starvation, ischemia, metabolism

## Abstract

Heart failure (HF) is an emerging epidemic with a high mortality rate. Apart from conventional treatment methods, such as surgery or use of vasodilation drugs, metabolic therapy has been suggested as a new therapeutic strategy. The heart relies on fatty acid oxidation and glucose (pyruvate) oxidation for ATP-mediated contractility; the former meets most of the energy requirement, but the latter is more efficient. Inhibition of fatty acid oxidation leads to the induction of pyruvate oxidation and provides cardioprotection to failing energy-starved hearts. One of the non-canonical types of sex hormone receptors, progesterone receptor membrane component 1 (*Pgrmc1*), is a non-genomic progesterone receptor associated with reproduction and fertility. Recent studies revealed that *Pgrmc1* regulates glucose and fatty acid synthesis. Notably, *Pgrmc1* has also been associated with diabetic cardiomyopathy, as it reduces lipid-mediated toxicity and delays cardiac injury. However, the mechanism by which *Pgrmc1* influences the energy-starved failing heart remains unknown. In this study, we found that loss of *Pgrmc1* inhibited glycolysis and increased fatty acid/pyruvate oxidation, which is directly associated with ATP production, in starved hearts. Loss of *Pgrmc1* during starvation activated the phosphorylation of AMP-activated protein kinase, which induced cardiac ATP production. *Pgrmc1* loss increased the cellular respiration of cardiomyocytes under low-glucose conditions. In isoproterenol-induced cardiac injury, *Pgrmc1* knockout resulted in less fibrosis and low heart failure marker expression. In summary, our results revealed that *Pgrmc1* ablation in energy-deficit conditions increases fatty acid/pyruvate oxidation to protect against cardiac damage via energy starvation. Moreover, *Pgrmc1* may be a regulator of cardiac metabolism that switches the dominance of glucose-fatty acid usage according to nutritional status and nutrient availability in the heart.

## 1. Introduction

Heart failure is an emerging epidemic, and patients with reduced ejection fraction rates have a mortality rate of >70% [1]. Despite extensive studies on the epidemiology and risk factors, the mortality rate of heart failure remains high [2]. Malnutrition is a known risk factor for myocardial damage [3]. Clinically, individuals are exposed to malnutrition-mediated cardiac risks during surgery, sepsis, and some serious diseases [4]. Currently used drugs for cardiomyopathy, such as angiotensin-converting enzyme inhibitors or beta blockers, reduce vasoconstriction and decrease the risk of death [5]. However, improving the function of the heart itself will provide a more fundamental breakthrough in the treatment of energy-starved heart failure. ATP production is mainly derived from fatty acid oxidation in the heart [6]. Heart failure with hypertension or ischemia is accompanied by decreased cardiac fatty acid oxidation [7]. Similarly, glucose oxidation, another pathway for ATP production, is also suppressed in heart failure [8]. As a failing heart lacks energy due to decreased glucose and fatty acid oxidation, targeting cardiac energy metabolism is the main research focus of many studies [9].

Although subtypes differ between sexes, the overall heart failure risk is comparable between men and women [10]. Some beneficial effects of androgen and estrogen on heart failure have been previously reported [11,12]. While synthetic progestin is considered to have deleterious effects, the influence of progesterone or canonical progesterone receptors in heart failure is neither beneficial nor deleterious [13]. One of the progesterone receptors, progesterone receptor membrane component 1 (*Pgrmc1*), has been reported to suppress obesity/diabetes-mediated cardiac lipotoxicity [14]. *Pgrmc1* is a non-canonical progesterone receptor associated with reproductive functions, such as decidualization [15] and female fertility [16]. Recent studies have revealed the metabolic function of *Pgrmc1*, beyond the reproductive relationships, in liver [17] and adipose tissue [18], focusing on the anabolism of glucose and lipids. Regulation of insulin, a major anabolic hormone, by *Pgrmc1* has also been reported in the pancreas [19]. Although *Pgrmc1*-related anabolisms have been extensively studied, the mechanism of *Pgrmc1*-related catabolism remains ambiguous. Furthermore, the regulation of cardiac health by *Pgrmc1* has been investigated only in the energy-enriched state in diabetes. In this study, we investigated how *Pgrmc1*-related catabolism affects cardiac health during energy starvation. Based on previous reports on the apoptosis and necrosis of cardiomyocytes during glucose starvation in vivo and in vitro [20,21], we used glucose starvation mouse models (72 h fasting) to mimic cardiac ischemia under physiological conditions in this study. Additionally, an adrenergic stimulation model using isoproterenol injection was introduced to induce energy starvation in the heart based on previous studies indicating lowered ATP production from ADP in the isoproterenol model [22]. Unlike the overnutrition state, *Pgrmc1* loss increased fatty acid and pyruvate oxidation in the heart during malnutrition. Our results indicated that maintenance of the major energy production pathway protected the *Pgrmc1*-ablated heart from energy starvation-induced injury.

## 2. Materials and Methods

### 2.1. Animals

Wild-type (WT) and *Pgrmc1* global knockout (PKO) littermate mice [23] (8-week-old; C57BL/6 background) were grown in a pathogen-free facility at Chungnam National University under a standard 12:12 h light:dark cycle and fed standard chow diet with water provided ad libitum. The mice were fasted to starvation, and unexpected deaths during the experiment were recorded to assess the survival rate. Isoproterenol (230 mg/kg, subcutaneous) was injected for two weeks to induce adrenergic heart damage. To observe cardiac pumping in WT and PKO mice, fluorescent dye-labeled (DyLight 680 antibody labeling kit, Thermo Scientific, Waltham, MA, USA, 53056) bovine serum albumin (BSA) was intravenously injected into the mice. After 1 h, the mice were anesthetized and placed in an in vivo imaging system (IVIS; FOBI, Vancouver, BC, Canada). A video was recorded to observe cardiac pumping. Images of cardiac contraction/relaxation were also captured. All animal experiments were approved by the Chungnam Facility Animal Care Committee (CNU-00606) and adhered to their ethical guidelines.

### 2.2. Gene Expression Omnibus (GEO) Datasets

Public datasets (GEO) were used to determine *PGRMC1* transcription levels in patients with cardiomyopathy. GSE29819 and GSE36961 datasets were selected, and all patients were included in the analysis.

### 2.3. Comprehensive Laboratory Animal Monitoring System (CLAMS)

CLAMS was used to assess the metabolic status of starved mice. Oxygen consumption (VO_2_) and carbon dioxide production (VCO_2_) rates were measured using an Oxymax system (Columbus Instruments, Columbus, OH, USA). Mice were placed at least 50 min before experiment for acclimation. The respiratory exchange ratio (RER) and respiratory quotient (RQ) were calculated as the ratio of VCO_2_ to VO_2_. The mice were fasted from midway through the light cycle to midway through the dark cycle.

### 2.4. RNA Isolation, Reverse Transcription, and Quantitative Reverse Transcription–Polymerase Chain Reaction (qRT-PCR)

RNA pellets were collected from the hearts of mice and H9c2 cells using TRIzol, chloroform, and isopropanol. RNA pellet was washed with ethanol and dissolved in diethyl pyrocarbonate-treated water. RNA concentration was measured, and the same RNA amounts for each sample were used for cDNA synthesis using an Excel RT Reverse transcriptase kit (SG-cDNAS100; Smartgene, Daejeon, Republic of Korea). Real-time PCR was carried out using specific primers (Table 1), Excel Taq Q-PCR Master Mix (SG-SYBR-500; Smartgene), and Stratagene Mx3000P (Agilent Technologies, Santa Clara, CA, USA) in a 96-well optical reaction plate. Negative controls containing water instead of the sample cDNA were used in each plate.

### 2.5. Western Blotting

Protein samples were resolved on 8–12% sodium dodecyl sulfate (SDS) polyacrylamide gels (running buffer: 25 mM Tris, 192 mM Glycine, 0.1% SDS, and D.W.). After electrophoresis, the gels were blotted onto a polyvinylidene difluoride membrane (IPVH 00010; Millipore, Burlington, MA, USA) at 350 mA for 1–2 h with the transfer buffer (25 mM Tris, 192 mM Glycine, and 20% (*v*/*v*) methanol). Membranes were blocked in 3% BSA and incubated with primary antibodies overnight at 4 °C. Membranes were washed thrice with TBS-T to remove the excess antibodies and incubated overnight at 4 °C with the following secondary antibodies: goat anti-rabbit IgG horseradish peroxidase (HRP) (Catalog #31460) and goat anti-mouse IgG HRP (Catalog #31430; Thermo Fisher Scientific, Waltham, MA, USA) antibodies. After washing thrice with TBS-T, immunoreactive proteins were observed with ECL solution (Eta C Ultra 2.0; Cyanagen, Bologna, Italy) using a ChemiDoc system (Fusion Solo, Vilber Lourmat, Eberhardzell, Germany).

The following primary antibodies were used: *PGRMC1* (13856; Cell Signaling Technology, Danvers, MA, USA), ribosomal protein lateral stalk subunit P0 (RPLP0; A13633; Abclonal, Woburn, MA, USA), poly(ADP ribose) polymerase (PARP; 9532; Cell Signaling Technology), C/EBP homologous protein (CHOP; #MA1-250; Invitrogen, Waltham, MA, USA), β-actin (sc-47778; Santa Cruz, Dallas, TX, USA), glycolysis antibody sampler kit (8337; Cell Signaling Technology), pAMPK, tAMPK (9957; Cell Signaling Technology), LC3B (L7543, Sigma-Aldrich, St. Louis, MO, USA), and α-tubulin (66031-1-Ig; Proteintech, Rosemont, IL, USA).

### 2.6. Blood and Plasma Measurements

For blood glucose measurement, the tail was snipped, and the blood glucose levels were measured using an Accu-Chek Active kit (Roche, Basel, Switzerland). During necropsy, blood was collected from the IVC. Plasma samples were analyzed to determine the levels of free fatty acids (FFAs; BM-FFA100, Biomax, Planegg, Germany), triglycerides (TGs; TG-1650, Fuji Film, Tokyo, Japan), and total cholesterol (TCHO; TCHO-1450).

### 2.7. Cell Culture

All the cell culture reagents were purchased from Welgene (Gyeongsan, Republic of Korea). H9c2 rat cardiomyocytes were maintained in Dulbecco’s modified Eagle’s medium (LM001-05; Welgene) supplemented with 5% (*v*/*v*) fetal bovine serum (FBS, Punjab, Pakistan), penicillin (100 U/mol), and streptomycin (100 μg/mL). To reflect the plasma profile of mice, cells were incubated with a low-glucose/fatty acid medium (500 mg/L glucose, 110 µM palmitic acid, 220 μM oleic acid) for 24 h. For *Pgrmc1* knockdown/overexpression experiments, cells were incubated with Opti-MEM (31985070; Gibco; without FBS) for 0.5 h and treated with the siRNA/plasmid and lipofectamine 2000 (11668027; Thermo Fisher Scientific). The siRNA sequence used was: 5′-CAGUUCACUUUCAAGUAUCA-U-3′. Medium containing FBS was later added after 6 h.

### 2.8. Cardiac Fibrosis Measurement

Tissues were fixed with neutral-buffered formalin, and trimmed tissues were washed with tap water. Tissues were subjected to serial dehydration and embedded in paraffin. The paraffin block was cut (5 μm) using a microtome, and the cut sections were attached to a silane-coated slide. Slides were immersed in xylene overnight and processed using a commercial kit (MST-100T; Biognost, Zagreb, Croatia), according to the manufacturer’s protocol, for Masson’s Trichrome staining. Regions of interest were observed under a light microscope.

### 2.9. Terminal Deoxynucleotidyl Transferase-Mediated dUTP Nick End-Labeling (TUNEL) Staining and Immunostaining

Frozen tissues were embedded in an optimal cutting temperature compound and cut (8 μm) using a cryostat. Slides were dried overnight and washed with TBS-T. TUNEL assay (11684795910; Roche, Basel, Switzerland) was performed according to the manufacturer’s protocol. After 4′,6-diamidino-2-phenylindole staining, the region of interest was observed under a fluorescence microscope. For immunostaining, frozen tissue slides were dried overnight and heated in oven (65 °C) for 10 min. Slides were immersed in distilled water and subsequently TBS-T. After blocking with 3% BSA, slides were incubated with primary antibody (CD31, ab56299; Abcam, Cambridge, UK) overnight at 4 °C. The next day, slides were washed with TBS-T and incubated with secondary antibody (A21202, Life Technologies, Carlsbad, CA, USA) for 4 h at room temperature. The region of interest was observed under a fluorescence microscope.

### 2.10. Statistical Analysis

Data are reported as the mean ± standard deviation. Differences between means were analyzed via Student’s *t*-test and one-way analysis of variance followed by Tukey’s multiple comparison test using the Graph Pad Software (GraphPad Inc., San Diego, CA, USA). Statistical significance was set at *p* < 0.05.

## 3. Results

### 3.1. PGRMC1 Expression Is Associated with Energy-Starved Cardiomyopathy

**Figure 1:**
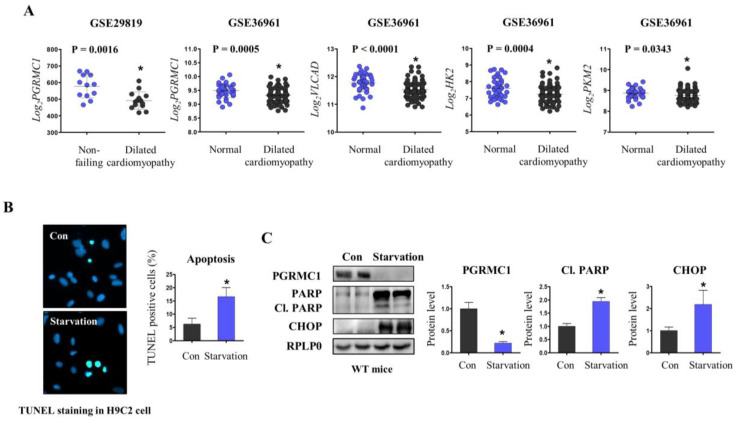
Progesterone receptor membrane component 1 (*PGRMC1*) expression is associated with energy-starved heart failure. Public Gene Expression Omnibus (GEO) datasets were used for analysis. Patients data for analyses were from 12 non-failing hearts and 14 dilated cardiomyopathy cases in one dataset (GSE29819) and from 39 normal and 106 hypertrophic cardiomyopathy cases in another dataset (GSE36961). (**A**) mRNA expression levels of *PGRMC1* (GSE29819 and GSE36961) and metabolic enzymes (GSE36961) were analyzed. (**B**) Terminal deoxynucleotidyl transferase-mediated dUTP nick end-labeling (TUNEL) immunostaining of H9C2 cells in a growth medium (Con; glucose 4500 mg/L, 5% fetal bovine serum (FBS)) and starvation medium (glucose 0 mg/L, 1% FBS). Notably, 4′,6-diamidino-2-phenylindole (DAPI) was used to stain the nucleus control. (**C**) Western blotting analysis and quantification of the expression levels of *PGRMC1*, poly(ADP ribose) polymerase (PARP), cleaved PARP (Cl. PARP), and C/EBP homologous protein (CHOP) in hearts of resting and 72-h-starved mice. Ribosomal protein lateral stalk subunit P0 (RPLP0) was used as an internal control. Five mice from each group were used for the experiment. Student’s *t*-test was used for analysis. Values represent the mean ± standard deviation (SD). * *p* < 0.05.

Using public clinical datasets, we collected data to investigate the relationship between *PGRMC1* expression and cardiomyopathy. In GSE29819, both ventricles from patients with dilated cardiomyopathy showed lower *PGRMC1* expression levels than those from non-failing donor hearts (Figure 1A). In GSE36961, the hearts of patients with dilated cardiomyopathy with left ventricular systolic dysfunction showed decreased *PGRMC1* expression levels compared to those of normal individuals (Figure 1A). Interestingly, the expression levels of key enzymes involved in fatty acid oxidation and glycolysis were lower in the hearts of patients with dilated cardiomyopathy (Figure 1A).

Through several in vitro and in vivo experiments, we attempted to delineate the effects of energy starvation on cardiomyocyte health. We induced energy starvation in H9C2 cardiomyocytes and mice via glucose starvation (glucose 0 mg/L, FBS 1%) and fasting (72 h), respectively. As shown in Figure 1B, cells under glucose starvation were predisposed to apoptotic cell death. Furthermore, hearts from mice under starvation (72 h) showed increased protein levels of apoptotic markers (cleaved PARP) and endoplasmic reticulum stress markers (CHOP) compared to those under resting conditions (Con) (Figure 1C). *PGRMC1* protein expression was markedly suppressed by fasting (Figure 1C). These results indicate that *PGRMC1* levels are closely related to energy starvation-induced cardiomyocyte injury.

### 3.2. Loss of PGRMC1 Maintains the Whole-Body Metabolism during Starvation

**Figure 2:**
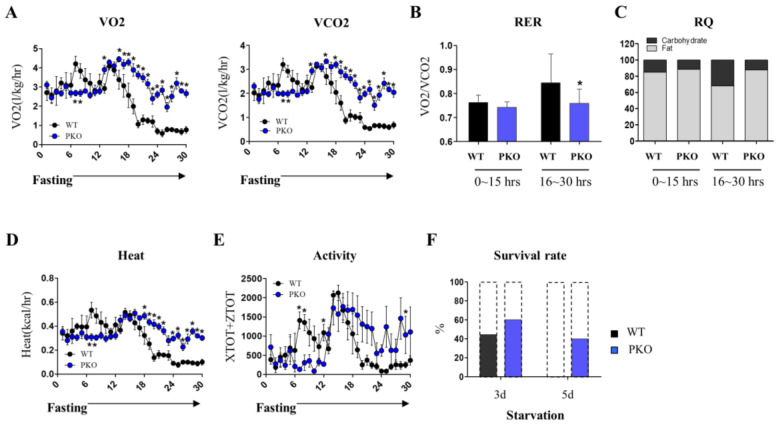
*Pgrmc1* knockout (PKO) protects the heart from energy starvation-induced metabolic suppression. (**A**) Oxygen consumption (VO_2_; l/kg/h) and carbon dioxide production (VCO_2_; l/kg/h). (**B**) Respiratory exchange ratio (RER) was calculated as VCO_2_/VO_2_. (**C**) Respiratory quotient (RQ) was calculated as the proportion of VCO_2_ to VO_2_. (**D**,**E**) Heat generation and activity measurements. Comprehensive lab animal monitoring system (CLAMS) was adopted for all assessments. Mice (wild-type (WT); n = 6, PKO; n = 8) were fasted during the tests. Student’s *t*-test was used for analysis. (**F**) Survival rate of WT (n = 9) and PKO (n = 5) mice during fasting. Mice under fasting died spontaneously. Values represent the mean ± SD. * *p* < 0.05.

Since there is no information on the physiological profile of PKO mice under starvation, we used CLAMS for comprehensive assessments. In CLAMS, VO_2_ levels were markedly reduced from 14 h fasting and reached baseline after 20 h fasting in WT mice. In contrast, VO_2_ levels were generally maintained at high levels in PKO mice during fasting. VCO_2_ levels showed a similar pattern as the VO_2_ levels. Levels of VCO_2_ markedly decreased after 14 h of fasting and reached baseline after 20 h of fasting in WT mice. In contrast, PKO mice maintained high VCO_2_ levels during fasting (Figure 2A). Additionally, the RER (VO_2_/VCO_2_) ratios were lower in PKO mice than in WT mice during prolonged fasting (Figure 2B). RQ calculation revealed that PKO mice are more likely to consume fat than glucose during prolonged fasting (Figure 2C). The heat production of PKO mice was highly maintained during fasting, notably from 14 h fasting, compared to that of WT mice (Figure 2D). The physical activity of PKO mice was also maintained during the prolonged fasting period, while that of WT mice was substantially diminished during the same period (Figure 2E). When mice were starved for a long period, some died unexpectedly due to an energy deficit. PKO mice were resistant to starvation-induced death compared to WT mice (Figure 2F). These results indicate that PKO mice are physiologically resistant to energy starvation.

### 3.3. Pgrmc1 Loss Increases Fatty Acid/Pyruvate Oxidation and Decreases Starvation-Induced Cardiac Injury

**Figure 3:**
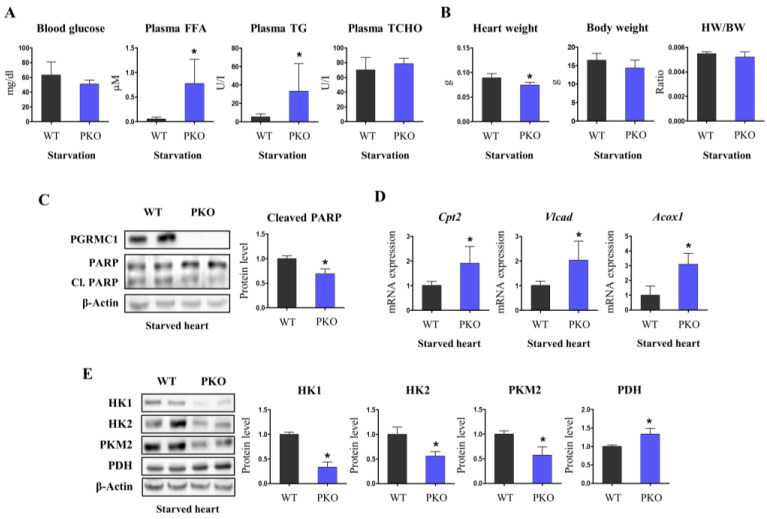
PKO increases fatty acid/pyruvate oxidation and restrains cardiac injury under energy starvation. (**A**) Levels of blood glucose (mg/dL), plasma free fatty acids (FFAs; µM), plasma triglycerides (TGs; U/I), and plasma total cholesterol (TCHO; U/I) in starved WT and PKO mice. (**B**) Heart weight (HW), body weight (BW), and HW/BW ratio in starved WT and PKO mice. (**C**) Western blotting analysis and quantification of the expression levels of *PGRMC1*, PARP, and Cl. PARP in the starved hearts of WT and PKO mice. β-Actin was used for an internal control. (**D**) mRNA expression levels of fatty acid oxidation enzymes in the starved hearts of WT and PKO mice. *Rplp0* was used as an internal control. (**E**) Western blotting analysis and quantification of the levels of glycolysis and pyruvate oxidation enzymes in the starved hearts of WT and PKO mice. β-Actin was used as an internal control. Mice used for the experiments: 8 (WT) and 4 (PKO). Student’s *t*-test was used for analysis. Values represent the mean ± SD. * *p* < 0.05.

To investigate how *Pgrmc1* will affect the heart under starvation, WT and PKO mice were starved for 72 h and exposed to cardiac malnutrition. Blood glucose levels were at baseline in both starved WT and PKO mice, showing no difference between the two groups (Figure 3A). Plasma lipid profiles increased in starved PKO mice. Notably, plasma FFA and TG levels were significantly higher in starved PKO mice than in starved WT mice (Figure 3A). Heart weight (HW) decreased in starved PKO mice, while the ratio of HW per body weight (BW) was similar (Figure 3B). Western blotting showed that starved PKO hearts had decreased cleaved PARP levels, which is an apoptotic marker, compared to starved WT hearts (Figure 3C). Concordantly, PKO hearts showed seemingly increased cardiac contractions in the IVIS using fluorescence (Figure S1).

Most hearts with hypertrophy or failure undergo metabolic alterations characterized by decreased fatty acid oxidation [24]. Fatty acid oxidation accounts for almost 70% of cardiac energy production [25]. PKO hearts under starvation conditions showed significantly increased expression levels of mitochondrial fatty acid oxidation enzymes (carnitine palmitoyltransferase 2 (*Cpt2*) and very long-chain acyl-CoA dehydrogenase (*Vlcad*)) and peroxisomal fatty acid oxidation enzyme (acyl-CoA oxidase 1 (*Acox1*)) compared to WT hearts under starvation conditions (Figure 3D). Glycolysis is a rapidly induced cardiac metabolism process associated with heart failure [26]. PKO hearts under starvation had markedly decreased protein levels related to glycolysis (hexokinase (HK)-1, HK2, and pyruvate kinase M2 (PKM2)) (Figure 3E). Glucose oxidation accelerates cardiac function recovery following myocardial injury [27]. Likewise, dichloroacetate, a pyruvate dehydrogenase (PDH) activator, increases myocardial efficiency [28]. Cardiac PDH was higher in PKO than in WT plants under starvation conditions (Figure 3E). These results indicate that starved PKO hearts increase their main energy production and fatty acid/pyruvate oxidation and do not need to be exposed to metabolic alterations.

**Figure 4:**
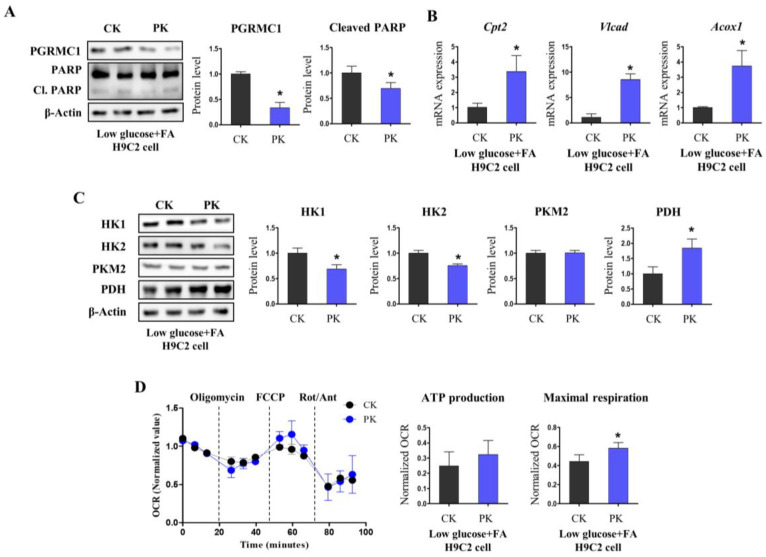
*Pgrmc1* knockdown in cardiomyocytes reduces cardiac damage with induction of fatty acid/glucose oxidation in a low-glucose medium supplemented with fatty acids. (**A**) Western blotting analysis and quantification of the levels of *PGRMC1*, PARP, and cleaved PARP in H9c2 cells treated with the control small interfering RNA (siRNA) (control knockdown, CK) and *Pgrmc1* siRNA (*Pgrmc1* knockdown, PK). β-Actin was used as an internal control. (**B**) mRNA expression levels of fatty acid oxidation enzymes in H9c2 cells treated with the control siRNA (CK) and *Pgrmc1* siRNA (PK). *Rplp0* was used as an internal control. (**C**) Western blotting analysis and quantification of the levels of hexokinase (HK)-1, HK2, pyruvate kinase M2 (PKM2), and pyruvate dehydrogenase (PDH) in H9c2 cells treated with the control siRNA (CK) and *Pgrmc1* siRNA (PK). β-Actin was used as an internal control. (**D**) Oxygen consumption rate (OCR) of CK and PK cells during mitochondrial stress test. ATP production and maximal respiration rates were calculated by changing the OCR after oligomycin and rotenone/antimycin (Rot/Ant) treatments, respectively. Cells were incubated in a medium containing low glucose (500 mg/L) and fatty acids (palmitic acid (110 µM)/oleic acid (220 µM)). All experiments were repeated at least three times. Student’s *t*-test was used for analysis. Values represent the means ± SD. * *p* < 0.05.

As plasma FFA levels were highly maintained in PKO mice, it should be tested whether these metabolic alterations are influenced by the levels of physiologically induced substrates. To limit the influential factors in vivo, we introduced H9c2 rat cardiomyocytes and knocked down *Pgrmc1* by siRNA. The cells were exposed to low glucose (500 mg/L) and fatty acids (palmitic acid (110 µM)/oleic acid (220 µM)). *PGRMC1* protein levels were lower in the PK (*Pgrmc1* knockdown) group than in the CK (control knockdown) group (Figure 4A). Cleaved PARP levels were lowered in PK group (Figure 4A). Metabolic alterations followed in vivo results. The mRNA expression levels of *Cpt2*, *Vlcad*, and *Acox1* were higher in the PK group than in the CK group (Figure 4B). The protein levels of HK1 and HK2 decreased in the PK group (Figure 4C). PDH levels increased in the PK group (Figure 4C). Collectively, in vitro *Pgrmc1* knockdown in low-energy cardiomyocytes induced fatty acid/pyruvate oxidation and decreased cellular injury. To investigate whether metabolic alterations in the PK group increased energy production compared to that in the CK group under energy deficit, we introduced a seahorse flux analyzer system to measure cellular respiration. H9c2 cells were knocked down and starved in a medium containing low glucose (500 mg/L) and fatty acids (palmitic acid (110 µM)/oleic acid (220 µM)). In the mitochondrial stress test, the PK group had a higher maximal respiration rate than that of the CK group (Figure 4D). We also measured the mitochondrial fusion/fission gene expression levels to assess the mitochondrial balance [29]. PKO hearts had a mildly increased fission gene (dynamin-related protein 1; Drp1) expression level compared to WT hearts (Figure S2A). These results confirm that fatty acid/pyruvate oxidation by PK increases energy production even under reduced glycolysis.

### 3.4. AMPK Activation Is Associated with Pgrmc1-Induced Metabolic Alteration in the Heart

**Figure 5:**
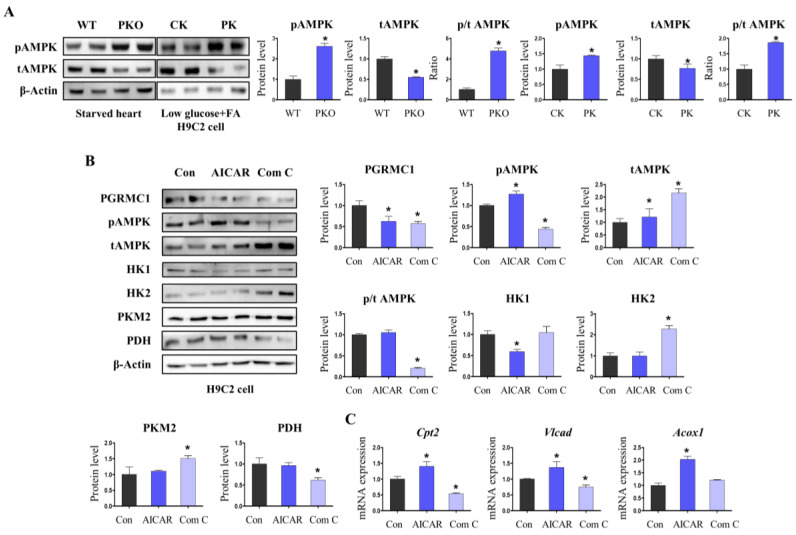
Loss of *Pgrmc1* increases the phosphorylation of AMP-activated protein kinase (AMPK) in the heart under energy starvation. (**A**) Western blotting analysis and quantification of the levels of pAMPK, tAMPK, and p/t AMPK in starved hearts of WT and PKO mice and H9c2 cells treated with the control siRNA (CK) and *Pgrmc1* siRNA (PK). β-Actin was used as an internal control. Mice used for experiments were eight (WT) and four (PKO) in number. Cells were incubated in a medium containing low glucose (500 mg/L) and FAs (palmitic acid (110 µM)/oleic acid (220 µM)). (**B**) Western blotting analysis and quantification of the levels of *PGRMC1*, pAMPK, tAMPK, p/t AMPK, HK1, HK2, PKM2, and PDH in H9c2 cells treated with AICAR (200 µM) and Com C (5 µM). β-Actin was used for an internal control. (**C**) mRNA expression levels of fatty acids oxidation enzymes in H9c2 cells treated with 5-aminoimidazole-4-carboxamide ribonucleotide (AICAR; 200 µM) and compound C (Com C; 5 µM). *Rplp0* was used as an internal control. All experiments were repeated at least three times. Student’s *t*-test was used for analysis. Values represent the mean ± SD. * *p* < 0.05.

We investigated the possible mechanism of metabolic alterations induced by *Pgrmc1*. AMPK is a multi-functional protein kinase involved in the oxidation and uptake of metabolites [30]. Western blotting revealed that starved PKO hearts had increased phosphorylated AMPK (pAMPK) levels and decreased total AMPK (tAMPK) levels. Starved PKO hearts showed a higher p/t AMPK ratio than WT hearts (Figure 5A). In H9c2 cells, PK cells showed higher pAMPK and lower tAMPK levels than CK cells. Concordantly, PK cells showed an increased p/t AMPK ratio compared to that in CK cells (Figure 5A).

Metabolic effects of AMPK activation and inactivation in cardiomyocytes were assessed. *PGRMC1* levels were not directly regulated by AMPK activation because treatments with 5-aminoimidazole-4-carboxamide ribonucleotide (AICAR; AMPK activator) and compound C (Com C; AMPK inactivator) suppressed *PGRMC1* expression. AMPK phosphorylation was increased by AICAR and decreased by Com C treatment (Figure 5B). HK1 levels were lowered by AICAR, whereas HK2 and PKM2 levels were increased by Com C. *PDH* levels were decreased by Com C (Figure 5B). In contrast, the expression levels of fatty acid oxidation enzymes were markedly increased by AICAR treatment (Figure 5C). Com C treatment decreased *Cpt2* and *Vlcad* expression levels (Figure 5C). In summary, AMPK activation was related to the induction of fatty acid/pyruvate oxidation and decreased glycolysis. As *Pgrmc1* loss increased AMPK activation and showed similar metabolic alterations to AMPK-activated cells, AMPK may be linked to metabolic modulation by *PGRMC1* in starved hearts.

### 3.5. Pgrmc1 Ablation Protects the Heart from Isoproterenol-Induced Damage

**Figure 6:**
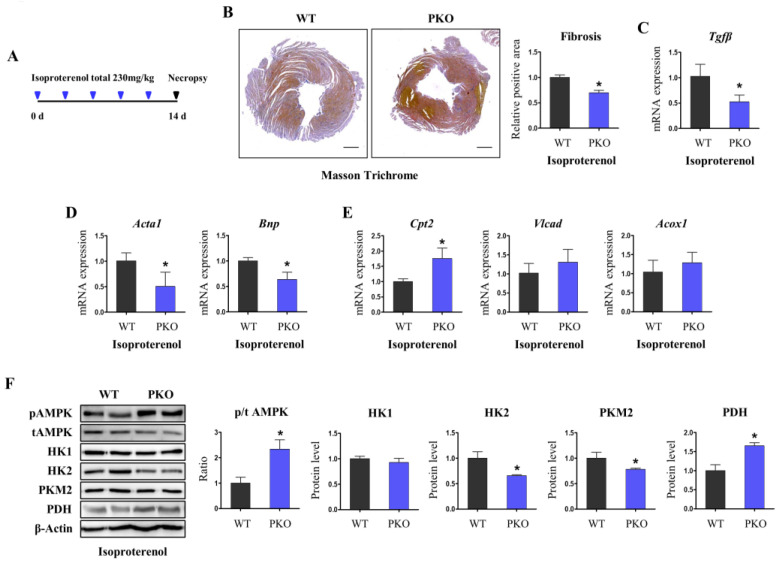
PKO protects against cardiac injury induced by isoproterenol treatment. (**A**) Experimental scheme for isoproterenol-induced cardiac injury. Isoproterenol was injected five times (total 230 mg/kg) via a subcutaneous injection for two weeks. (**B**) Masson’s Trichrome staining of isoproterenol-injected WT and PKO hearts. Cardiac fibrosis was calculated by the ratio of blue area to brown area. Scale bar: 600 µm. (**C**) mRNA expression levels of transforming growth factor (*Tgf*)-*β* in isoproterenol-injected WT and PKO hearts. *Rplp0* was used as an internal control. (**D**) mRNA expression levels of heart failure markers, i.e., actin alpha 1 (*Acta1*) and brain natriuretic peptide (*Bnp*), in isoproterenol-injected WT and PKO hearts. *Rplp0* was used as an internal control. (**E**) mRNA expression levels of fatty acid oxidation enzymes, carnitine palmitoyltransferase 2 (*Cpt2*), very long-chain acyl-CoA dehydrogenase (*Vlcad*), and acyl-CoA oxidase 1 (*Acox1*), in isoproterenol-injected WT and PKO hearts. *Rplp0* was used as an internal control. (**F**) Western blotting analysis and quantification of the levels of pAMPK, tAMPK, p/t AMPK, HK1, HK2, PKM2, and PDH in isoproterenol-injected hearts of WT and PKO mice. β-Actin was used as an internal control. Mice used for experiments were six (isoproterenol-WT) and six (isoproterenol-PKO) in number. Student’s *t*-test was used for analysis. Values represent the mean ± SD. * *p* < 0.05.

We introduced isoproterenol cardiac injury model according to previous studies [31,32]. Mice were injected with isoproterenol (five times, total 230 mg/kg, 14 days) and sacrificed (Figure 6A). Masson’s trichrome staining revealed that isoproterenol-WT hearts showed large positive areas with fibrosis (Figure 6B). In contrast, isoproterenol-PKO hearts showed decreased fibrotic areas compared with WT hearts (Figure 6B). Transforming growth factor-beta mRNA expression levels decreased in isoproterenol-PKO hearts (Figure 6C). As heart failure markers, mRNA expression levels of actin alpha 1 and brain natriuretic peptide were decreased in isoproterenol-PKO hearts compared to those in WT hearts (Figure 6D). In metabolic assessments, isoproterenol-PKO hearts showed higher levels of fatty acid oxidation enzymes (*Cpt2*) than isoproterenol-WT hearts (Figure 6E). Furthermore, isoproterenol-PKO hearts had decreased glycolysis enzyme levels and increased PDH levels. Additionally, isoproterenol-PKO hearts showed an increased p/t ratio of AMPK (Figure 6F). Hence, isoproterenol-PKO hearts had altered cardiac metabolism, such as fasting-PKO cardiac metabolism, increased fatty acid/pyruvate oxidation and AMPK phosphorylation, and decreased glycolysis. Maintenance of the ATP-producing pathway, i.e., fatty acid/pyruvate oxidation, may provide cardioprotection under ischemic injury.

## 4. Discussion

Ischemic heart failure is prevalent worldwide [33]. Beyond traditional surgery, various methods using protein, cell, and gene therapeutics have been suggested for treatment [34]. Notably, several regulators of cardiac metabolism have been identified [35]. The heart relies heavily on long-chain fatty acids and utilizes glucose low-proportionally for energy production in the normal state [36]. Both fatty acid oxidation and glucose oxidation produce acetyl-CoA, which directly participates in the tricarboxylic acid cycle and electron transport chain and accounts for 95% of myocardial ATP production [7]. In failing hearts, fatty acid availability substantially affects the myocardial function and efficiency [37]. Additionally, pyruvate oxidation, leading to the production of acetyl-CoA from glucose-derived pyruvate, is limited in heart failure, resulting in impaired ATP production [7]. Thus, failing hearts are etiologically or resultantly associated with impaired energy production via fatty acid/pyruvate oxidation.

During cellular stress, AMPK phosphorylation downregulates fatty acid synthesis but upregulates fatty acid oxidation [38]. Although fatty acid oxidation itself can suppress pyruvate oxidation, AMPK activation increases glycolysis and pyruvate oxidation. Due to its diverse effects, whether AMPK improves or deteriorates the cardiac health may differ according to the physiological state of the patient [39]. AMPK has been reported to increase overall ATP production to respond to the energy demand and provide tolerance against cardiac ischemia [40]. When the hearts were exposed to fasting or isoproterenol-induced energy starvation, PKO increased AMPK phosphorylation. Catabolic activation by PKO differed according to metabolic pathways; fatty acid and pyruvate oxidation increased, but glycolysis decreased. Fatty acid oxidation takes place predominantly in the mitochondria and peroxisomes in less magnitude [41]. Mitochondrial fatty acid oxidation enzymes [42], namely *Cpt2* and *Vlcad*, and the peroxisomal fatty acid oxidation enzyme [43] *Acox1* increased in PKO hearts. The high availability of plasma fatty acids in PKO may influence catabolic processes. However, exposure to the same amount of fatty acids in in vitro experiment also increased fatty acid oxidation in PK cells. Conversely, *Pgrmc1*-overexpressing (POE) cells exhibited decreased fatty acid oxidation (Figure S3). Hence, an increase in the fatty acid oxidation pathway affects cardiac energy metabolism in PKO. Paradoxically, PKO hearts have decreased levels of glycolytic enzymes, hexokinases, and pyruvate kinase but increased PDH [44]. When cells are exposed to the same amounts of glucose and fatty acids, PK cells still increase pyruvate oxidation but suppress glycolysis. Similarly, POE cells showed a mild increase in glycolysis (Figure S3). We speculated that the lactate source must be induced to increase pyruvate substrate and pyruvate dehydrogenase in limited sources from glycolytic products. Our results (data not shown) also showed the induction of lactate dehydrogenase in starved PKO hearts. Further studies on the regulation of lactate metabolism by *Pgrmc1* should be performed. Glycolysis only accounts for <10% [45], while the oxidation of fatty acids (50–70%) [46] and pyruvate (20–40%) [7] comprises the majority of cardiac ATP production. Hence, starved PKO hearts may have increased overall ATP production. Mechanistically, PKO hearts showed increased AMPK phosphorylation, and AMPK inhibitor (Com C) treatment resulted in the opposite cardiac metabolism pattern compared to that of PKO. In line with this, AMPK activator (AICAR) treatment showed a cardiac metabolism pattern similar to that of PKO. Concordantly, PKO-altered cardiac energy metabolism may be linked to AMPK phosphorylation during cardiac injury. We also measured the cardiac autophagy, as AMPK is an autophagy promoter [47], but observed significantly down-regulated LC3B levels in PKO hearts. As *Pgrmc1* is an autophagy promoter [48], cardiac autophagy was mainly affected by *Pgrmc1* compared to AMPK. This is in accordance with our results, as autophagy is up-regulated in ATP-depleted and ischemic hearts [49].

We insist on the interpretation of conflicting metabolic alterations and functions of PKO hearts in light of a previous study. In our previous study, PKO hearts in diabetic conditions showed increased TG and fatty acyl-CoA accumulation [14], leading to lipotoxicity. However, TG deposits play an ATP-providing role [50], and fatty acyl CoA is directly related to oxidative phosphorylation in the heart [51,52]. In contrast to overnutrition hearts, the large pool of lipids in PKO can be the ATP pool for energy-deficient hearts. Additionally, in our previous study, cardiac glycolysis was induced only in overnutrition PKO and slightly decreased in normal PKO hearts [14]. In the energy-deficient state, glycolysis was significantly decreased in PKO hearts. In contrast, fatty acid oxidation was decreased in normal and overnutrition PKO hearts [14] but increased in malnutrition PKO hearts. We concluded that cardiac metabolic alteration by *Pgrmc1* depends on glucose availability. In re-fed and diabetic mice, blood glucose levels were approximately 200 mg/dL [14], which were higher than those in starved mice (approximately 60 mg/dL). *Pgrmc1* may be a physiological switch that regulates the preference of cardiac substrates for ATP production depending on the body’s nutrition. In energy-deficit conditions, *Pgrmc1* reduces oxidation of fatty acids/pyruvates, thereby limiting ATP production in the heart.

The failing heart possesses a nearly 30% ATP volume [53] and reduces the ATP-supplementing flux from the reserve (creatine kinase) by 50% compared to the normal heart [54]. ATP depletion in the failing heart directly leads to contractile dysfunction because continuous ATP production/turnover is necessary for cardiac function [24]. Fatty acid oxidation is the major cardiac ATP-producing pathway, but it suppresses glucose oxidation, as per the Randle cycle [55]. Since glucose oxidation is a much more efficient ATP-production and less-oxygen-consuming pathway than fatty acid oxidation [28], its activation is therapeutically effective in a failing heart [56]. The fatty acid oxidation inhibitor etomoxir has been reported to exert cardioprotective effects by switching from energy metabolism to glucose oxidation [57,58]. However, adverse effects of fatty acid oxidation inhibition can also be observed in experimental/clinical reports [59,60]. Based on our results, *Pgrmc1* inhibition increases both fatty acid and pyruvate oxidation and improves overall ATP production during energy starvation. Therefore, improvement in ATP-production via a *Pgrmc1* inhibitor can be used as a novel therapeutic approach for energy-starved failing hearts. Additionally, PKO hearts reduced CD31 abundance in immunostaining (Figure S2C). This result is of clinical importance, as CD31 levels are markedly observed in the necrotic myocardium of deceased patients under ischemic heart disease [61]. Furthermore, CD31 blockade reduces damage in ischemia/reperfusion heart injury [62]. As *Pgrmc1* promotes cellular processes of microvascular endothelial cells of the brain [63], further study is expected regarding *Pgrmc1* and the cardiovascular system.

## Figures and Tables

**Table 1:** Primers used for qRT-PCR.

Gene Name	Upper Primer (5′-3′)	Lower Primer (5′-3′)	Species
*Cpt2*	CAG CAC AGC ATC GTA CCC A	TCC CAA TGC CGT TCT CAA AAT	Mouse
*Vlcad*	TAT CTC TGC CCA GCG ACT TT	TGG GTA TGG GAA CAC CTG AT	Mouse
*Acox1*	TTG GAA ACC ACT GCC ACA TA	AGG CAT GTA ACC CGT AGC AC	Mouse
*Tgfβ*	GAC GTC ACT GGA GTT GTA CG	GGT TCA TGT CAT GGA TGG TG	Mouse
*Anp*	CCA TAT TGG AGC AAA TCC TGT G	CGG CAT CTT CTC CTC CAG GT	Mouse
*Bnp*	GGG AGA ACA CGG CAT CAT TG	ACA GCA CCT TCA GGA GAT CCA	Mouse
*Mfn2*	GCC AGC TTC CTT GAA GAC AC	GCA GAA CTT TGT CCC AGA GC	Mouse
*Drp1*	AGA AAA CTG TCT GCC CGA GA	GCT GCC CTA CCA GTT CAC TC	Mouse
*Cpt2*	ACT AAG AGA TGC TCC GAG GC	GCA GAG CAT ACA AGT GTC GG	Rat
*Vlcad*	TGA CCC TGC CAA GAA TGA CT	GTC ATG CAT GCC CAC AAT CT	Rat

## Data Availability

The data presented in this study are available on request from the corresponding author.

## Supplementary Materials

The following supporting information can be downloaded at: https://www.mdpi.com/article/10.3390/cells12050752/s1, Figure S1. IVIS monitoring of WT and PKO mice. Figure S2. Cardiac autophagy, mitochondrial fusion/fission, and vascularization enzyme levels. Figure S3. Influence of *Pgrmc1* overexpression in cardiac metabolism.

## References

[1] Groenewegen A., Rutten F.H., Mosterd A., Hoes A.W. (2020). Epidemiology of Heart Failure. Eur. J. Heart Fail..

[2] Roger V.L. (2021). Epidemiology of Heart Failure: A Contemporary Perspective. Circ. Res..

[3] Arikawa R., Kanda D., Ikeda Y., Tokushige A., Sonoda T., Anzaki K., Ohishi M. (2021). Prognostic impact of malnutrition on cardiovascular events in coronary artery disease patients with myocardial damage. BMC Cardiovasc. Disord..

[4] Webb J.G., Kiess M.C., Chan-Yan C.C. (1986). Malnutrition and the heart. Can. Med. Assoc. J..

[5] Shah A., Gandhi D., Srivastava S., Shah K.J., Mansukhani R. (2017). Heart Failure: A Class Review of Pharmacotherapy. P T Peer-Rev. J. Formul. Manag..

[6] Grynberg A., Demaison L. (1996). Fatty Acid Oxidation in the Heart. J. Cardiovasc. Pharmacol..

[7] Lopaschuk G.D., Karwi Q.G., Tian R., Wende A.R., Abel E.D. (2021). Cardiac Energy Metabolism in Heart Failure. Circ. Res..

[8] Jaswal J.S., Keung W., Wang W., Ussher J.R., Lopaschuk G.D. (2011). Targeting fatty acid and carbohydrate oxidation—A novel therapeutic intervention in the ischemic and failing heart. Biochim. Biophys. Acta (BBA)-Mol. Cell Res..

[9] Heggermont W.A., Papageorgiou A.P., Heymans S., van Bilsen M. (2016). Metabolic Support for the Heart: Complementary Therapy for Heart Failure?. Eur. J. Heart Fail..

[10] Lam C.S.P., Arnott C., Beale A.L., Chandramouli C., Hilfiker-Kleiner D., Kaye D.M., Ky B., Santema B.T., Sliwa K., Voors A.A. (2019). Sex differences in heart failure. Eur. Heart J..

[11] Güder G., Allolio B., Angermann C.E., Störk S. (2011). Androgen Deficiency in Heart Failure. Curr. Heart Fail. Rep..

[12] Iorga A., Umar S., Ruffenach G., Aryan L., Li J., Sharma S., Motayagheni N., Nadadur R.D., Bopassa J.C., Eghbali M. (2018). Estrogen rescues heart failure through estrogen receptor Beta activation. Biol. Sex Differ..

[13] Hermsmeyer R.K., Thompson T.L., Pohost G.M., Kaski J.C. (2008). Cardiovascular Effects of Medroxyprogesterone Acetate and Progesterone: A Case of Mistaken Identity?. Nat. Clin. Pract. Cardiovasc. Med..

[14] Lee S.R., Heo J.H., Jo S.L., Kim G., Kim S.J., Yoo H.J., Lee K.-P., Kwun H.-J., Shin H.-J., Baek I.-J. (2021). Progesterone receptor membrane component 1 reduces cardiac steatosis and lipotoxicity via activation of fatty acid oxidation and mitochondrial respiration. Sci. Rep..

[15] Russo N., Kaplan-Kahn E.A., Wilson J., Criss A., Burack J.A. (2021). Choices, challenges, and constraints: A pragmatic examination of the limits of mental age matching in empirical research. Dev. Psychopathol..

[16] McCallum M.L., Pru C.A., Niikura Y., Yee S.-P., Lydon J.P., Peluso J.J., Pru J.K. (2016). Conditional Ablation of Progesterone Receptor Membrane Component 1 Results in Subfertility in the Female and Development of Endometrial Cysts. Endocrinology.

[17] Lee S.R., Choi W.-Y., Heo J.H., Huh J., Kim G., Lee K.-P., Kwun H.-J., Shin H.-J., Baek I.-J., Hong E.-J. (2020). Progesterone increases blood glucose via hepatic progesterone receptor membrane component 1 under limited or impaired action of insulin. Sci. Rep..

[18] Furuhata R., Kabe Y., Kanai A., Sugiura Y., Tsugawa H., Sugiyama E., Hirai M., Yamamoto T., Koike I., Yoshikawa N. (2020). Progesterone receptor membrane associated component 1 enhances obesity progression in mice by facilitating lipid accumulation in adipocytes. Commun. Biol..

[19] Zhang M., Robitaille M., Showalter A.D., Huang X., Liu Y., Bhattacharjee A., Willard F.S., Han J., Froese S., Wei L. (2014). Progesterone Receptor Membrane Component 1 Is a Functional Part of the Glucagon-Like Peptide-1 (Glp-1) Receptor Complex in Pancreatic Beta Cells. Mol. Cell Proteomics.

[20] Wanka H., Lutze P., Albers A., Golchert J., Staar D., Peters J. (2021). Overexpression of Transcripts Coding for Renin-b but Not for Renin-a Reduce Oxidative Stress and Increase Cardiomyoblast Survival under Starvation Conditions. Cells.

[21] Xu X., Pacheco B.D., Leng L., Bucala R., Ren J. (2013). Macrophage migration inhibitory factor plays a permissive role in the maintenance of cardiac contractile function under starvation through regulation of autophagy. Cardiovasc. Res..

[22] El-Demerdash E., Awad A.S., Taha R.M., El-Hady A.M., Sayed-Ahmed M.M. (2005). Probucol attenuates oxidative stress and energy decline in isoproterenol-induced heart failure in rat. Pharmacol. Res..

[23] Lee S.R., Kwon S.W., Kaya P., Lee Y.H., Lee J.G., Kim G., Lee G.-S., Baek I.-J., Hong E.-J. (2018). Loss of progesterone receptor membrane component 1 promotes hepatic steatosis via the induced de novo lipogenesis. Sci. Rep..

[24] Doenst T., Nguyen T.D., Abel E.D. (2013). Cardiac Metabolism in Heart Failure: Implications Beyond Atp Production. Circ. Res..

[25] Lionetti V., Stanley W.C., Recchia F.A. (2011). Modulating fatty acid oxidation in heart failure. Cardiovasc. Res..

[26] Fillmore N., Levasseur J.L., Fukushima A., Wagg C.S., Wang W., Dyck J.R.B., Lopaschuk G.D. (2018). Uncoupling of glycolysis from glucose oxidation accompanies the development of heart failure with preserved ejection fraction. Mol. Med..

[27] Li T., Xu J., Qin X., Hou Z., Guo Y., Liu Z., Wu J., Zheng H., Zhang X., Gao F. (2017). Glucose oxidation positively regulates glucose uptake and improves cardiac function recovery after myocardial reperfusion. Am. J. Physiol. Metab..

[28] Tran D.H., Wang Z.V., Heather L.C., Pates K.M., Atherton H.J., Cole M.A., Ball D.R., Evans R.D., Glatz J.F., Luiken J.J. (2019). Glucose Metabolism in Cardiac Hypertrophy and Heart Failure. J. Am. Heart Assoc..

[29] Dorn G.W. (2016). Mitochondrial Fission/Fusion and Cardiomyopathy. Curr. Opin. Genet. Dev..

[30] Zaha V.G., Young L.H. (2012). AMP-Activated Protein Kinase Regulation and Biological Actions in the Heart. Circ. Res..

[31] Brooks W.W., Conrad C.H. (2009). Isoproterenol-induced myocardial injury and diastolic dysfunction in mice: Structural and functional correlates. Comp. Med..

[32] Grimm D., Elsner D., Schunkert H., Pfeifer M., Griese D., Bruckschlegel G., Muders F., Riegger G.A., Kromer E.P. (1998). Development of Heart Failure Following Isoproterenol Administration in the Rat: Role of the Renin-Angiotensin System. Cardiovasc. Res..

[33] Nordlie M.A., Wold L.E., Kloner R.A. (2005). Genetic contributors toward increased risk for ischemic heart disease. J. Mol. Cell. Cardiol..

[34] Choi D., Hwang K.-C., Lee K.-Y., Kim Y.-H. (2009). Ischemic heart diseases: Current treatments and future. J. Control Release.

[35] Fillmore N., Mori J., Lopaschuk G.D. (2014). Mitochondrial Fatty Acid Oxidation Alterations in Heart Failure, Ischaemic Heart Disease and Diabetic Cardiomyopathy. Br. J. Pharmacol..

[36] Polak-Iwaniuk A., Harasim-Symbor E., Gołaszewska K., Chabowski A. (2019). How Hypertension Affects Heart Metabolism. Front. Physiol..

[37] Tuunanen H., Engblom E., Naum A., Någren K., Hesse B., Airaksinen J., Nuutila P., Iozzo P., Ukkonen H., Opie L.H. (2006). Free Fatty Acid Depletion Acutely Decreases Cardiac Work and Efficiency in Cardiomyopathic Heart Failure. Circulation.

[38] Hardie D.G., Pan D.A. (2002). Regulation of fatty acid synthesis and oxidation by the AMP-activated protein kinase. Biochem. Soc. Trans..

[39] Dyck J.R., Lopaschuk G.D. (2006). Ampk Alterations in Cardiac Physiology and Pathology: Enemy or Ally?. J. Physiol..

[40] Arad M., Seidman C.E., Seidman J.G. (2007). Amp-Activated Protein Kinase in the Heart: Role during Health and Disease. Circ. Res..

[41] Stanley W.C., Recchia F.A., Lopaschuk G.D. (2005). Myocardial Substrate Metabolism in the Normal and Failing Heart. Physiol. Rev..

[42] Knottnerus S.J.G., Bleeker J.C., Wüst R.C.I., Ferdinandusse S., Ijlst L., Wijburg F.A., Wanders R.J.A., Visser G., Houtkooper R.H. (2018). Disorders of mitochondrial long-chain fatty acid oxidation and the carnitine shuttle. Rev. Endocr. Metab. Disord..

[43] Zeng J., Deng S., Wang Y., Li P., Tang L., Pang Y. (2017). Specific Inhibition of Acyl-Coa Oxidase-1 by an Acetylenic Acid Improves Hepatic Lipid and Reactive Oxygen Species (Ros) Metabolism in Rats Fed a High Fat Diet. J. Biol. Chem..

[44] Depre C., Rider M.H., Hue L. (1998). Mechanisms of control of heart glycolysis. JBIC J. Biol. Inorg. Chem..

[45] Allard M.F., Schonekess B.O., Henning S.L., English D.R., Lopaschuk G.D. (1994). Contribution of oxidative metabolism and glycolysis to ATP production in hypertrophied hearts. Am. J. Physiol. Circ. Physiol..

[46] Fukushima A., Lopaschuk G.D. (2016). Cardiac fatty acid oxidation in heart failure associated with obesity and diabetes. Biochim. Biophys. Acta (BBA)-Mol. Cell Biol. Lipids.

[47] Li Y., Chen Y. (2019). Ampk and Autophagy. Adv. Exp. Med. Biol..

[48] Zhu X., Ji M., Han Y., Guo Y., Zhu W., Gao F., Yang X., Zhang C. (2017). *PGRMC1*-dependent autophagy by hyperoside induces apoptosis and sensitizes ovarian cancer cells to cisplatin treatment. Int. J. Oncol..

[49] Gustafsson A.B., Gottlieb R.A. (2009). Autophagy in Ischemic Heart Disease. Circ. Res..

[50] Saddik M., Lopaschuk G.D. (1991). Myocardial triglyceride turnover and contribution to energy substrate utilization in isolated working rat hearts. J. Biol. Chem..

[51] Borutaite V., Mildaziene V., Ivanoviene L., Kholodenko B., Toleikis A., Praskevicius A. (1989). The Role of Long-Chain Acyl-Coa in the Damage of Oxidative Phosphorylation in Heart Mitochondria. FEBS Lett..

[52] Liu G.X., Hanley P.J., Ray J., Daut J. (2001). Long-Chain Acyl-Coenzyme a Esters and Fatty Acids Directly Link Metabolism to K(Atp) Channels in the Heart. Circ. Res..

[53] Ingwall J.S., Weiss R.G. (2004). Is the Failing Heart Energy Starved? On Using Chemical Energy to Support Cardiac Function. Circ. Res..

[54] Weiss R.G., Gerstenblith G., Bottomley P.A. (2005). Atp Flux through Creatine Kinase in the Normal, Stressed, and Failing Human Heart. Proc. Natl. Acad. Sci. USA.

[55] Hue L., Taegtmeyer H. (2009). The Randle cycle revisited: A new head for an old hat. Am. J. Physiol. Metab..

[56] Bersin R.M., Wolfe C., Kwasman M., Lau D., Klinski C., Tanaka K., Khorrami P., Henderson G.N., de Marco T., Chatterjee K. (1994). Improved hemodynamic function and mechanical efficiency in congestive heart failure with sodium dichloroacetate. J. Am. Coll. Cardiol..

[57] Schmidt-Schweda S., Holubarsch C. (2000). First Clinical Trial with Etomoxir in Patients with Chronic Congestive Heart Failure. Clin. Sci..

[58] Schmitz F., Rösen P., Reinauer H. (1995). Improvement of Myocardial Function and Metabolism in Diabetic Rats by the Carnitine Palmitoyl Transferase Inhibitor Etomoxir^®^. Horm. Metab. Res..

[59] Holubarsch C.J.F., Rohrbach M., Karrasch M., Boehm E., Polonski L., Ponikowski P., Rhein S. (2007). A double-blind randomized multicentre clinical trial to evaluate the efficacy and safety of two doses of etomoxir in comparison with placebo in patients with moderate congestive heart failure: The ERGO (etomoxir for the recovery of glucose oxidation) study. Clin. Sci..

[60] O’Connor R.S., Guo L., Ghassemi S., Snyder N.W., Worth A.J., Weng L., Kam Y., Philipson B., Trefely S., Nunez-Cruz S. (2018). The CPT1a inhibitor, etomoxir induces severe oxidative stress at commonly used concentrations. Sci. Rep..

[61] Kondo T., Takahashi M., Yamasaki G., Sugimoto M., Kuse A., Morichika M., Nakagawa K., Sakurada M., Asano M., Ueno Y. (2022). Immunohistochemical analysis of CD31 expression in myocardial tissues from autopsies of patients with ischemic heart disease. Leg. Med..

[62] Woodfin A., Voisin M.-B., Nourshargh S. (2007). PECAM-1: A Multi-Functional Molecule in Inflammation and Vascular Biology. Arter. Thromb. Vasc. Biol..

[63] Xu X., Ruan X., Ju R., Wang Z., Yang Y., Cheng J., Gu M., Mueck A.O. (2022). Progesterone Receptor Membrane Component-1 May Promote Survival of Human Brain Microvascular Endothelial Cells in Alzheimer’s Disease. Am. J. Alzheimer’s Dis. Other Dement..

