# Supplementary material for: PGRMC1 Ablation Protects from Energy-Starved Heart Failure by Promoting Fatty Acid/Pyruvate Oxidation
Source: Cells. 2023 Feb 27;12(5):752. doi: 10.3390/cells12050752 (PMC10000468; doi:10.3390/cells12050752)
Supplement: Supplementary file 1 [file cells-12-00752-s001.zip › cells-2207057-supplementary.pdf]

Supplementary Materials

PGRMC1 ablation protects from energy-starved heart failure by promoting fatty acid/pyruvate oxidation

Sang R. Lee[1,2], Moeka Mukae[1], Kang Joo Jeong[1], Se Hee Park[2], Hi Jo Shin[2], Sang Woon Kim[2], Young Suk Won[2], Hyo-Jung Kwun[1], In-Jeoung Baek[3] and Eui-Ju Hong[1*]

[1]College of Veterinary Medicine, Chungnam National University, Daejeon 34134, Republic of Korea
[2]Laboratory Animal Resource Center, Korea Research Institute for Biology and Biotechnology, Cheongju 28116, Republic of Korea
[3]Department of Convergence Medicine, University of Ulsan College of Medicine, Asan Medical Center, Seoul 05505, Republic of Korea;

[*]Address correspondence to:

Eui-Ju Hong, DVM, PhD

College of Veterinary Medicine, Chungnam National University

99 Daehak-ro, Yuseong-gu, Daejeon 34134, Korea

Phone: +82-42-821-6781; Fax: +82-42-821-8903; Email: ejhong@cnu.ac.kr

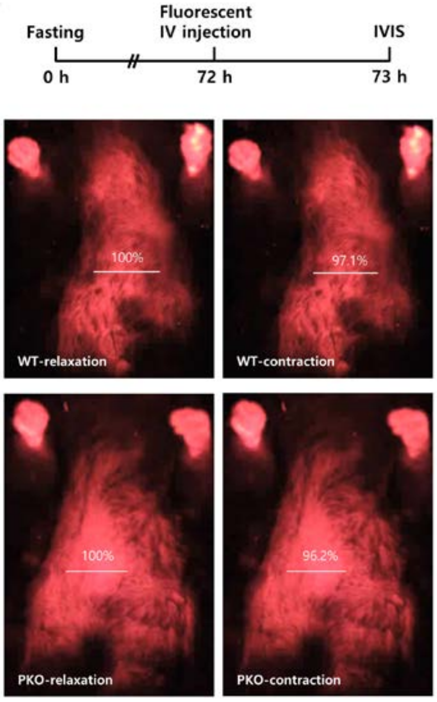

Figure S1. Cardiac diameter during contraction and relaxation measured by in situ fluorescence. Mice were fasted for 72 h and intravenously injected with fluorescent labeled-bovine serum albumin (BSA). After 1 h, hearts of mice were monitored under in vivo imaging system (IVIS).

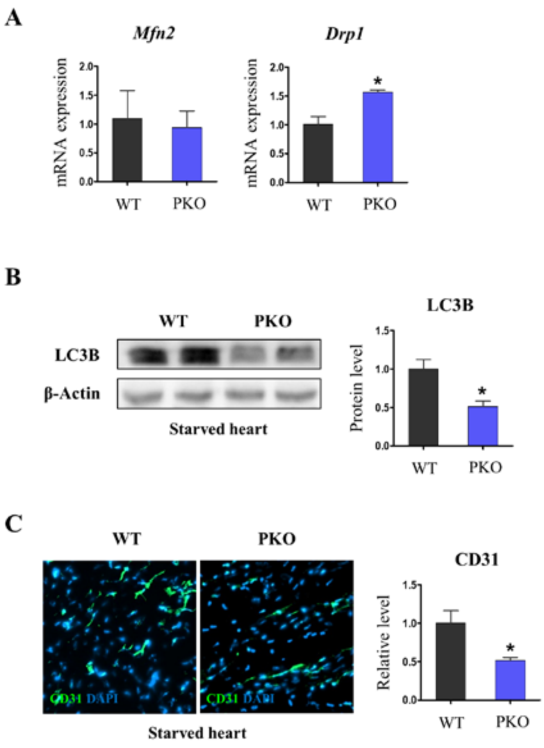

Figure S2. Cardiac gene levels related to autophagy, mitochondrial fusion/fission, and vascularization. (A) mRNA expression levels of *Mfn2* and *Drp1* in hearts of starved WT and PKO mice. *Rplp0* was used for an internal control. (B) Western blot analysis and quantification of LC3B in hearts of starved WT and PKO mice. β-Actin was used for an internal control. (C) Immunostaining of CD31 (green) in hearts of starved WT and PKO mice. DAPI (blue) was used for a nucleus control. Mice used for the experiments: 8 (WT) and 4 (PKO). Student's t-test was used for analysis. Values represent the mean ± SD. *$p < 0.05$.

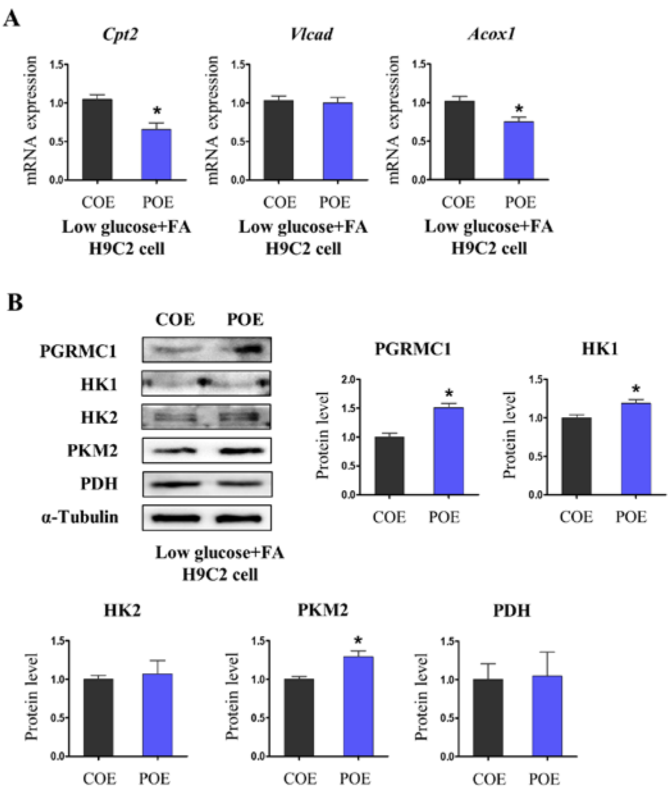

Figure S3. Pgrmc1 overexpression (POE) mildly modulates cardiac metabolism in H9c2 cells. (A) mRNA expression levels of fatty acids oxidation enzymes in control overexpressed (COE) and Pgrmc1 overexpressed (POE) H9c2 cells. *Rplp0* was used for an internal control. (B) Western blot analysis and quantification of PGRMC1, HK1, HK2, PKM2, and PDH in control overexpressed (COE) and Pgrmc1 overexpressed (POE) H9c2 cells. α-Tubulin was used for an internal control. Cells were incu-bated in medium containing low glucose (500 mg/l) and fatty acids (palmitic acid 110 μM/oleic acid 220 μM). All experiments were repeated at least three times. Student’s t-test was used for analysis. Values represent means ± SD. *, $p<0.05$.
